# Genomic and Clinical Profile of Hypervirulent *Klebsiella pneumoniae* (ST23-K1) Liver Abscesses: Experience with Patients at a Romanian University Hospital

**DOI:** 10.3390/pathogens15020237

**Published:** 2026-02-20

**Authors:** Dragoș Ștefan Lazăr, Elena Nedu, Adina-Alexandra Nanu, Maria-Irina Fediuc, Maria Alexandra Malciolu-Nica, Maria Nica, Mihaela Oprea, Oana-Claudia Albu, Laura-Ioana Popa, Daniela Andreea Blidaru, Simin Aysel Florescu

**Affiliations:** 1 Department of Infectious Diseases, “Carol Davila” University of Medicine and Pharmacy, 020021 Bucharest, Romania; dragos.lazar@umfcd.ro (D.Ș.L.); adina.nanu@drd.umfcd.ro (A.-A.N.); maria-irina.fediuc@rez.umfcd.ro (M.-I.F.); maria.nica@umfcd.ro (M.N.); daniela-andreea.blidaru0825@rez.umfcd.ro (D.A.B.); simin.florescu@umfcd.ro (S.A.F.); 2 “Dr. Victor Babes” Clinical Hospital of Infectious and Tropical Diseases, 030303 Bucharest, Romania; alexandra.malciolu@spitalulbabes.ro; 3 ”Cantacuzino” National Military Medical Institute for Research and Development, 050096 Bucharest, Romania; oprea.mihaela@cantacuzino.ro (M.O.); albu.oana@cantacuzino.ro (O.-C.A.); popa.laura@cantacuzino.ro (L.-I.P.)

**Keywords:** hypervirulent *Klebsiella pneumoniae*, liver abscess, endogenous endophthalmitis ST23-K1, genomic surveillance, virulence determinants

## Abstract

**Introduction**: Hypervirulent *Klebsiella pneumoniae* (hvKp) is an emerging global pathogen that causes severe liver abscesses and metastatic infections. Despite rising concerns regarding multidrug-resistant convergence, molecular data in Romania remain limited. This study characterizes the epidemiological, clinical, and genomic profiles of hvKp liver abscesses in a tertiary hospital, aiming to describe the local virulence landscape and inform clinical management. **Results**: This study characterizes 15 cases of hvKp liver abscesses in a tertiary hospital. The cohort was predominantly male, with high rates of type 2 diabetes. Although clinical presentation was severe, featuring complications like endogenous endophthalmitis, the outcomes following prolonged antibiotic therapy were generally favorable. Phenotypically, 93.3% of isolates retained a wild-type susceptible profile, while a single ESBL-positive case highlighted the risk of resistance convergence. Genomic sequencing confirmed the presence of the ST23-K1 pandemic lineage carrying key virulence determinants (*rmpA*, *iuc*, and *peg-344*). Crucially, cgMLST analysis revealed genetic heterogeneity, suggesting sporadic community acquisition rather than a clonal nosocomial outbreak. **Conclusions**: These findings represent the first detailed molecular description of hvKp in Romania, confirming the local circulation of high-risk ST23 clones and underscoring the necessity for early detection and surveillance.

## 1. Introduction

*Klebsiella pneumoniae* (Kp) is a clinically significant Gram-negative pathogen, known for causing both healthcare-associated and community-acquired infections. Historically, Kp has been primarily linked to pneumonia, urinary tract infections, and bacteremia, especially in immunocompromised or hospitalized patients [1].

Kp is classified into two main virulence phenotypes: classic *K. pneumoniae* (cKp) and hypervirulent *K. pneumoniae* (hvKp) [2]. The classic phenotype exhibits significant diversity in capsule (K) types and a high tendency for resistance to cephalosporins, carbapenems, and colistin, contributing to the global spread of multiple multidrug-resistant (MDR) lineages (notably ST258, ST15, ST11, and ST307) [3].

The hypervirulent subtype was first reported in Taiwan in 1986 and was found to be capable of causing severe community-acquired infections in otherwise healthy individuals, including liver abscess, endophthalmitis, meningitis, and necrotizing fasciitis. Over the past three decades, its incidence has been increasing; while it was initially reported predominantly in Asia, it is now emerging as a global health concern [4,5,6]. This syndrome is frequently associated with diabetes mellitus and specific capsular types (especially K1, K2, K5, K20, K54, K57, and KN1) that predispose affected individuals to severe metastatic complications [7,8]. Since the condition is considered to be primarily community-acquired, it was initially postulated that its source is intestinal and it spreads through translocation—a fact later proven in several studies [9,10].

HvKp strains are defined by the combined expression of multiple virulence factors that confer a markedly increased capacity for tissue invasion and immune evasion. These factors include the hypermucoviscous phenotype, a thick polysaccharide capsule, and the production of siderophores such as aerobactin [11]. Capsular serotype K1 is most strongly implicated in hypervirulence, especially in the appearance of liver abscesses, alongside genetic determinants such as *magA*, *rmpA*, *iucA*, and *peg-344* [11,12]. The presence of plasmid-encoded virulence elements underscores the potential for horizontal gene transfer, enabling the dissemination of hypervirulent traits across different clonal backgrounds [4]. Infections caused by hvKp typically occur in previously healthy individuals, with a median patient age of 50–60 years, and only rare cases have been described in pediatric populations [3,13].

Clinically, pyogenic liver abscess represents the most common manifestation, but hvKp can also give rise to abscesses in the brain, spleen, and lungs, as well as severe invasive syndromes such as meningitis, endogenous endophthalmitis, thrombophlebitis, and pneumonia [2,3,4,8]. Early reports indicated that these strains were largely susceptible to commonly used antimicrobials [2,4]; however, since 2014, more evidence has highlighted an alarming trend toward the convergence of hypervirulence and multidrug resistance [14,15,16]. The hvKp isolates that produce carbapenemases, including KPC, VIM, NDM, and OXA-48 enzymes, are being increasingly detected worldwide [4,6,7]. Moreover, high-risk nosocomial clones such as ST11, ST147, ST258, and ST307 have demonstrated the ability to acquire hypervirulent phenotypes, further complicating treatment options and infection control [6,7].

The combination of enhanced pathogenicity and extensive antimicrobial resistance presents a critical public health threat with the potential to cause severe, disseminated infections that are difficult to treat. Therefore, a comprehensive understanding of the epidemiology, molecular mechanisms of virulence, and resistance determinants is essential to inform effective diagnostic approaches, antimicrobial stewardship, and prevention strategies aimed at mitigating the impact of hypervirulent and MDR Kp [2,4,6].

To date, no study in Romania has specifically described the presence and molecular profile of hvKp. Most of the available research has focused on the evolution of MDR Kp strains [17,18]. However, as early as 2014, the presence of this phenotype was reported in our country through case studies on liver abscesses, sepsis, and endophthalmitis [19,20].

The present study attempts to integrate the epidemiological, clinical, and molecular characteristics of a series of cases diagnosed with hvKp liver abscesses in a tertiary infectious diseases center over a period of two and a half years.

## 2. Materials and Methods

### 2.1. Study Design, Setting, and Isolate Selection

We conducted a descriptive, retrospective, observational study between 1 January 2023 and 30 June 2025 at the “Dr. Victor Babeș” Clinical Hospital of Infectious and Tropical Diseases (VBH) in Bucharest, Romania. This tertiary infectious disease hospital provides care for patients with severe infections or those caused by pathogens resistant to conventional treatments.

Our study included patients with liver abscesses identified via imaging criteria (ultrasound or CT scan), whose Kp etiology was microbiologically confirmed. For each enrolled case, we recorded demographic data, relevant comorbidities, the clinical course during hospitalization at VBH, complications, the patient’s geographic origin, and comprehensive molecular sequencing results, including capsular type and the presence of the *magA*, *rmpA*, and *iucABCD* genes.

The data were collected from the hospital’s information system (InfoWorld), gathered from clinical departments, the pharmacy, and the microbiology laboratory, and processed using Microsoft Excel.

### 2.2. Microbiological Evaluation

Microbiological analysis was performed in the Microbiology Laboratory of the Diagnostic Department of VBH on clinical samples obtained from the Infectious Diseases and Intensive Care Units. All Kp strains were isolated from patient blood cultures, which were then subjected to antimicrobial susceptibility testing.

Identification of the Kp isolates was performed using an automated system based on matrix-assisted laser desorption/ionization–time-of-flight mass spectrometry (MALDI-TOF MS; Bruker Daltonics, Bremen, Germany).

Antimicrobial susceptibility testing was performed using the standardized Kirby–Bauer disk diffusion method (Oxoid, Basingstoke, UK) for the following antibiotics: ampicillin, amoxicillin/clavulanic acid, piperacillin/tazobactam, cefuroxime, ceftriaxone, ceftazidime, cefepime, imipenem, meropenem, amikacin, gentamicin, levofloxacin, ciprofloxacin, trimethoprim/sulfamethoxazole, ceftazidime/avibactam, ceftolozane/tazobactam, imipenem/relebactam, and meropenem/vaborbactam.

Additionally, MICRONAUT-S test plates (Bruker Daltonics, Bremen, Germany) were utilized for quantitative susceptibility testing of Gram-negative bacteria and the qualitative phenotypic detection of carbapenemases. These assays were performed using cation-adjusted Mueller–Hinton broth (CAMHB). Minimum inhibitory concentrations (MICs) were interpreted according to the EUCAST guidelines (2023/2024). The results were read using a Tecan plate reader (Tecan, Männedorf, Switzerland) and analyzed with the MICRONAUT6 software.

Phenotypic evaluation of hyperviscosity (string test) was not performed, as it is not a routine procedure in the VBH laboratory. Consequently, the classification of hvKp was based strictly on molecular criteria.

Multidrug resistance was defined according to the international consensus criteria proposed by Magiorakos et al. (2012) [21]; namely, non-susceptibility to at least one agent in three or more antimicrobial classes.

From a total of 15 *Klebsiella pneumoniae* isolates obtained from positive blood cultures, five isolates were selected for whole-genome sequencing. These isolates were chosen based on clinical severity and epidemiological relevance, including cases with metastatic complications (such as endogenous endophthalmitis), a fatal outcome, or recurrent disease. The aim of sequencing was to characterize isolates with the highest suspected hypervirulence potential, rather than to provide a statistically representative sample of the entire cohort.

### 2.3. Whole-Genome Sequencing Methodology

These five Kp isolates were subsequently submitted to the National Institute of Research & Development for Microbiology & Immunology “Cantacuzino” (NIRDMI) for whole-genome sequencing (WGS). Genomic DNA extraction was performed using the Maxwell^®^ RSC Cultured Cells DNA Kit (Promega, Madison, WI, USA), according to the manufacturer’s protocol. DNA libraries were then prepared utilizing the Nextera XT DNA Library Preparation Kit (Illumina, San Diego, CA, USA), and sequencing was executed on an Illumina MiSeq instrument using the MiSeq Reagent Kit v3 (600 cycles).

### 2.4. Software used for WGS data analysis

Bioinformatic analysis was mainly performed using the commercial software SeqSphere+ (Ridom, Münster, Germany) version 10.5.5, currently called MBioSeq Ridom Typer (Bruker, Germany) [22]. The raw reads were assembled using SKESA version 2.4.0 (de novo assembly; GPL v3; source code unmodified) [23].

The assemblies produced good quality draft 5.5–5.6 Mbp Kp genomes, consisting of 66–96 contigs, with N50 values ranging from 185,558 to 272,097 and a mean coverage per base of 65–93x.

Virulence factors were detected using the Virulence Factor Database (VFDB) and AMRFinder Plus, with the latter also being used to search for antimicrobial resistance determinants, while plasmids were predicted using the MOB-suite package from Chromosome and plasmid overview tool [24]. Sequence type (ST) and complex type (CT) were determined using multilocus sequence typing (MLST; 7-locus scheme) and core genome MLST (cgMLST; 2 358-locus scheme), respectively, in order to determine the genomic relatedness of the isolates [25].

The assembled genomes were also analyzed on the Pathogenwatch platform (https://pathogen.watch/), where Kleborate (version 3.1.3) was used to determine the capsular serotype and virulence-associated genes, and a virulence score (ranging from 0 to 5) was assigned to indicate the hypervirulence potential [26].

The raw sequencing reads have been uploaded to the European Nucleotide Archive (ENA) sequence database under accession number PRJEB91646.

### 2.5. Statistical Analysis

Data were analyzed using descriptive statistics. Categorical variables (e.g., sex and drainage type) are presented as frequencies and percentages, while continuous variables (e.g., age, maximum abscess size, and duration of antibiotic therapy) are reported as medians with ranges. Due to the observational design and the limited sample size, the analysis was restricted to descriptive methods, and no inferential statistical tests were performed.

### 2.6. Ethical Considerations

In accordance with Romanian regulations, all patients admitted to the hospital are given the opportunity to provide or decline consent for participation in research activities as part of the administrative paperwork completed at admission. All data were handled in compliance with institutional ethical standards and national data protection laws. The study was approved by the local ethics committee of the “Dr. Victor Babeș” Clinical Hospital (No. 10635/17.06.2025).

## 3. Results

### 3.1. Patient Characteristics, Clinical Presentation, and Complications

We analyzed 15 patients hospitalized between January 1, 2023, and June 30, 2025, who were diagnosed with Kp-induced liver abscesses. In 2023, 2024, and the first 6 months of 2025, three, four, and eight patients were admitted, respectively. The demographic characteristics of this cohort revealed a predominance of male patients (10 out of 15), with a median age of 66.5 years (IQR: 64–71) and a body mass index (BMI) of 28.4 (IQR: 24.6–31.0).

The majority of patients presented with comorbidities, with the most frequent being diabetes mellitus (*n* = 8; 53.3%), arterial hypertension (*n* = 7; 46,7%), dyslipidemia (*n* = 4), and hypothyroidism (*n* = 3). One patient (Case 8) had a pancreatic neoplasm complicated by pulmonary metastases and pleurisy. Conversely, no underlying comorbidities were identified in a single patient (Case 14). Case 15 presented with recurrent liver abscesses; this admission marked the third episode following previous treatments in 2021 and 2022.

Clinically, the inaugural symptom of fever accompanied by chills occurred in 13 cases, with eight of these also presenting with hepatalgia or diffuse abdominal pain, and four cases reporting significant asthenia. In two cases, patients presented with diarrhea as an inaugural symptom. The average period between symptom onset and hospitalization was 5.8 days.

Management involved percutaneous drainage in two patients and surgical drainage in two. For 11 patients, drainage was deemed unsuitable due to the patient’s condition, an inaccessible lesion location, or small abscess size. Three of these patients developed endogenous endophthalmitis, and two developed sepsis. Notably, Case 9 suffered a fulminant course; diagnosed with endophthalmitis, the patient progressed rapidly to septic shock and multiple organ dysfunction syndrome, passing away within 52 h of admission. The remaining patients showed a favorable clinical response to antibiotic therapy, with imaging revealing significant lesion reduction (*n* = 12) or complete resolution (*n* = 2).

Table 1 presents the main characteristics of the study patients.

From an imaging perspective, where abdominal ultrasound initially raised suspicion of a hepatic abscess, hepatomegaly was identified in 10 cases and hepatic steatosis was demonstrated in 13 cases, predominantly of a low grade. Contrast-enhanced CT examination confirmed the suspected diagnosis. In the majority of cases, we encountered multiple hepatic abscesses, except for four cases where they were solitary. The dimensions of the abscesses were highly variable, ranging from 13 × 21 mm to 140 × 100 mm, with most being located in the right hepatic lobe (13 of 15), one in the left lobe, and in one case in both lobes. In only one case did we observe multiple abscesses in the left hepatic lobe (Case 2) and, in another, in both hepatic lobes (Case 6). These characteristics are summarized in Table 2.

Endogenous endophthalmitis (EE), recognized as one of the most critical complications described in the literature, arises when an infectious agent disseminates hematogenously, proliferates within the choroid and subsequently infiltrates the retina, ultimately spreading into the vitreous [27]. In our study, EE was documented in three of the analyzed cases (Cases 9, 12, and 15). Case 9 succumbed to the infection approximately 52 h post-admission. For the other two patients, topical antibiotic therapy (including imipenem, netilmicin/dexamethasone, and tropicamide) was administered during their hospitalization. Despite this treatment, their clinical ocular evolution was unsatisfactory, leading to their subsequent referral to an ophthalmology hospital for vitrectomy. Notably, in Case 15, who was experiencing their third episode of liver abscesses, EE was only diagnosed during this current event and had not been reported during the preceding two disease episodes.

**Figure 1:**
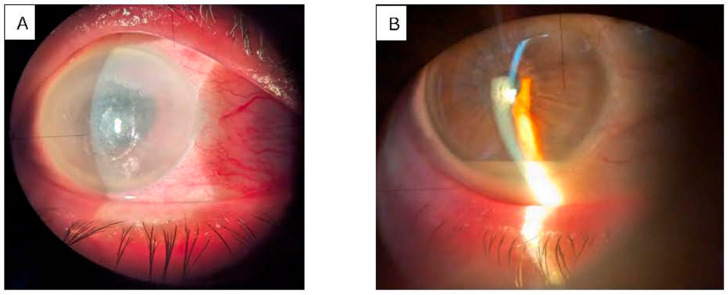
Slit lamp examination of in a patient with a hepatic abscess (Case 12): (**A**) right eye with red, painful eye, corneal edema, and unresponsive mydriasis; (**B**) left eye with “quiet eye” and hypopyon. Photo: Malciolu-Nica M.A., VBH Collection. Patient has consented to the publication of these photographs.

**Figure 2:**
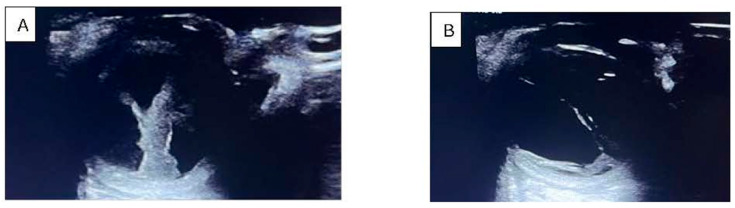
B-scan ultrasound showing (**A**) inflammatory membranes, dense hyperechogenic lesions, and inflamed periocular tissue; (**B**) tractional inflammatory membranes. The posterior segment cannot be visualized because it is obscured by the vitritis. Photo: Malciolu-Nica M.A. Patient has consented to the publication of these photographs.

**Figure 3:**
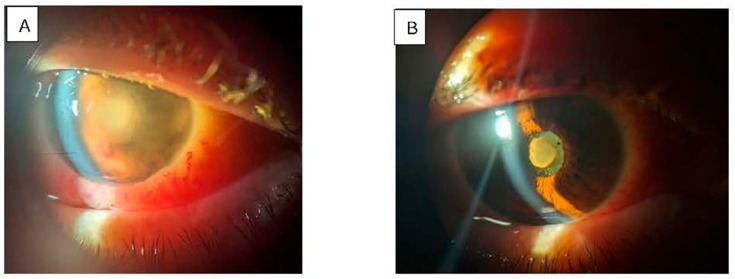
Slit lamp examination of a patient with sepsis and hepatic abscess (Case 15): (**A**) left eye with intense hyperemia of the conjunctiva and serous chemosis, dense inflammation in the anterior chamber, and corneal edema; (**B**) right eye with discrete hyperemia of the conjunctiva, and inflammatory pupillary membrane. Photo: Malciolu-Nica M.A., VBH Collection. Patient has consented to the publication of these photographs.

Clinically, patients presented with a unilateral red, painful eye, accompanied by decreased visual acuity, photophobia, and floaters (Figure 1 and Figure 2). Ocular examination revealed conjunctival injection, corneal edema, hypopyon, anterior chamber cells, iritis, and vitritis. When the posterior segment could not be visualized, B-scan ultrasonography was utilized to identify vitritis, as well as retinal or choroidal lesions (Figure 3). Figure 1, Figure 2 and Figure 3 illustrate the characteristic features observed in patients with endogenous endophthalmitis (EE).

### 3.2. Geographical Distribution of Cases

**Figure 4:**
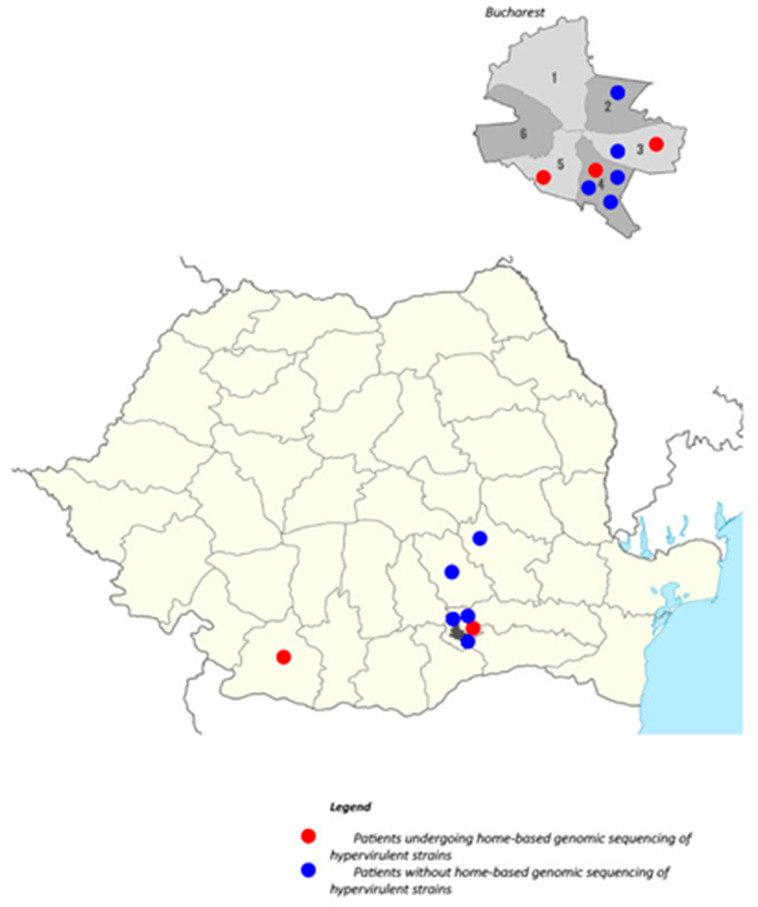
Geographical distribution of cases. Red circles indicate cases with sequenced isolates (WGS performed), while blue circles indicate non-sequenced cases. The map reflects referral patterns to the tertiary center and does not imply population-based incidence.

VBH is a center dedicated to treating complex cases from both Bucharest and the southeastern region of Romania. An analysis of the geographical distribution revealed that eight cases originated in Bucharest, four in the peri-urban area, and three in other southern regions of the country. Notably, of the eight cases within Bucharest, seven were located in the southern part of the city. Regarding the five cases selected for sequencing, three originated from Bucharest, one from the peri-urban zone, and one from the southern region of the country (Craiova). Figure 4 illustrates the geographical distribution of these cases; the five red circles denote the cases where hvKp sequencing was performed, while the blue circles represent non-sequenced cases. Even though most cases came from Bucharest, the high population density and the small number of observed cases prevent us from concluding that there is a higher concentration in this city.

### 3.3. Biomarker Analysis

Table 3 summarizes the hematological, biochemical, and inflammatory parameters recorded between hospital admission and day 21 of treatment.

Upon admission, patients exhibited leukocytosis and neutrophilia, accompanied by mild monocytosis and moderate thrombocytopenia and lymphopenia. A consistent finding among the liver abscess patients in our cohort was hypo- or aneosinophilia. Hepatic involvement was moderate, with alanine aminotransferase (ALT) and aspartate aminotransferase (AST) levels approximately twice the upper limit of normal. Regarding the inflammatory response, we observed fibrinogen values double the upper normal limit and significant elevations in C-reactive protein (CRP) levels.

For procalcitonin, generally considered a reliable marker for evaluating and monitoring bacterial infections, admission values demonstrated substantial variability. This variability is likely attributable to pre-existing renal insufficiency in some patients. Specifically, the mean admission procalcitonin was 51.4 ng/mL (SD 89.76), with a median of 12.59 ng/mL (IQR = 47). Due to this heterogeneity, we opted to exclude this parameter from further analysis.

Following 21 days of treatment, normalization of complete blood count values, as well as hepatic and renal parameters, was observed; however, a mild inflammatory syndrome persisted.

### 3.4. Antimicrobial Susceptibility Profiles

Antimicrobial susceptibility testing was successfully performed for 14 of the 15 enrolled cases; data were unavailable for Case 8 as the isolate was identified at an external facility. Multidrug resistance was exclusively based on acquired resistance patterns, in accordance with international definitions, and intrinsic resistance characteristics were not considered for multidrug resistance classification. Overall, the majority of isolates (*n* = 12) displayed a wild-type susceptibility profile, retaining high susceptibility to most antimicrobial classes.

Specifically,

- Carbapenems: 100% susceptibility was observed for imipenem, meropenem, and ertapenem;
- Aminoglycosides: All isolates were susceptible to amikacin and gentamicin;
- Fluoroquinolones: Susceptibility to ciprofloxacin was high, with only one isolate (Case 7) showing intermediate resistance based on MIC determination.

Only one isolate in the study (Case 4) showed a multidrug-resistant profile. This strain was resistant to several cephalosporins and to aztreonam, but it remained susceptible to carbapenems and β-lactam/β-lactamase inhibitor combinations. Overall, the resistance pattern was compatible with an Extended-Spectrum Beta-Lactamase-like phenotype (ESBL-like) according to standardized international terminology [21].

Phenotypic confirmatory testing for ESBL production using a clavulanate-based synergy assay was performed and supported the presence of an ESBL-like phenotype in Case 4. However, as whole-genome sequencing was not available for this isolate, the specific ESBL-encoding gene could not be identified.

### 3.5. Whole-Genome Sequencing Results and Molecular Epidemiology

Five *hvKp* strains (from Cases 1, 9, 12, 14, and 15) were sequenced at NIRDMI. All isolates belonged to the ST23-K1 lineage. Each genome exhibited a maximum Kleborate virulence score of 5, harboring a comprehensive suite of hypervirulence-associated genes, including *rmpA/rmpA2*, *rmpC*, *rmpD*, *magA*, *peg-344*, and the siderophore systems *iucABCD*, *iutA*, and *iroBCDN*. Furthermore, each isolate contained a large multireplicon IncFIB/IncHI1B plasmid (~224–235 Kbp) that carries these key virulence determinants.

The cgMLST analysis assigned the five isolates to distinct complex types, indicating genetic heterogeneity rather than a clonal cluster. Regarding antimicrobial resistance, no acquired resistance determinants were detected in the sequenced isolates; only the blaSHV-1 gene, which is intrinsic to *Klebsiella pneumoniae*, was identified and is reported for completeness, without contributing to the MDR phenotype or having specific epidemiological significance in this cohort.

The results of these molecular determinations are summarized in Table 4.

## 4. Discussion

Although hvKp has been thoroughly documented in East Asia—particularly in Taiwan, China, and South Korea—data originating from Europe are more limited and fragmented [6,28]. In a study published in 2025, using data available up to December 2023, it was found that hvKp was identified in 15.2% of Kp isolates worldwide. Based on the data available at that time, hvKp accounted for 29.9% of isolates in Asia, 9.56% of isolates in North America, and 6.67% of isolates in Europe [29].

In European countries such as France, Spain, Italy, and Poland, most reports describe sporadic cases or small clusters involving K1/ST23 or K2/ST86 strains, primarily associated with liver abscesses and metastatic complications, including endophthalmitis [30,31,32,33]. Overall, the incidence remains considerably lower than in Asia [34]. The regional risk of hvKp spread is likely underestimated due to diagnostic challenges and the absence of routine virulence testing. However, no confirmed sustained transmission within the EU/EEA has been reported to date [28]. Global surveillance has increasingly reported the convergence of hypervirulence and carbapenem resistance, notably within the ST23-K1 lineage, presenting a critical therapeutic challenge [35,36]. Kp is well known for its remarkable ability to survive on environmental surfaces and in water for prolonged periods. Importantly, it can persist in biofilms and contaminated wastewater, where it remains viable and capable of horizontal gene transfer. This includes the exchange of plasmids encoding carbapenem resistance (such as *bla*KPC-2 and *bla*NDM-1) and virulence determinants with other Klebsiella strains or Enterobacteriaceae, thereby facilitating the emergence of highly resistant clones even in the absence of direct patient contact [34,35].

By contrast, in Romania, there is a clear gap in published research specifically addressing hvKp. To date, most national studies have concentrated on multidrug-resistant and carbapenemase-producing isolates, without a systematic approach to evaluating virulence markers [37,38]. Thus, to our knowledge, the present study represents the first detailed description in Romania of the molecular features of hvKp isolates and their correlation with clinical outcomes.

Our study highlights the distinct clinical and epidemiological profile of Kp liver abscesses (KLAs) in a Romanian tertiary care setting; that is, a male predominance (67%), advanced median age, and BMI of around 28.

A central finding in this cohort is the high burden of comorbidities, present in 93.3% of patients. Type 2 diabetes mellitus (T2DM) was the most frequent underlying condition (53.3%). The link between diabetes mellitus and KLAs has been highlighted by several authors, which is likely due to the impaired neutrophil phagocytosis and enhanced bacterial growth caused by hyperglycemia [39,40,41]. Additionally, hepatic steatosis may also play a role in the development of the disease—a finding that we observed in all the patients studied and agrees with other data in the literature [41].

The clinical picture often began with fever and abdominal discomfort, and imaging frequently revealed hepatomegaly and hepatic steatosis. The majority of the abscesses were located in the right lobe of the liver, as also observed by other authors. However, in our study, only 3 of 15 cases had solitary abscesses, whereas the literature generally reports a predominance of solitary lesions [40,42].

The severity varied, including a fulminant course (endophthalmitis with septic shock leading to death within 52 h) and several complications (endogenous endophthalmitis, septic pulmonary emboli, and hepatic vein thrombosis). Therapeutically, the majority of our patients (73%) were managed conservatively with antibiotics alone as drainage was often contraindicated due to lesion size or location. Despite this, the outcomes were largely favorable, with significant lesion reduction in most cases. This supports the efficacy of prolonged antibiotic therapy in cases where infection control via drainage is not feasible, provided that the strain is susceptible.

Endogenous endophthalmitis (EE) emerged as a rare but severe complication in our KLA cohort. EE occurred in 3 of 15 cases (Cases 9, 12, and 15), including one fulminant demise within 52 h of admission (Case 9). The other two cases required ophthalmologic intervention (vitrectomy) after suboptimal responses to initial topical therapy, underscoring EE’s potential for rapid progression despite systemic management.

Geographically, the clustering of cases in Bucharest and southern Romania reflects the referral patterns of our institution (VBH) rather than a definitive epidemiological hotspot. However, the confirmed presence of the ST23-K1 lineage in sequenced cases suggests this hypervirulent clone is circulating in our region.

Biomarker analysis reveals a pattern typical of severe bacterial infection; in particular, leukocytosis and neutrophilia with mild monocytosis, thrombocytopenia, lymphopenia, and fluctuating procalcitonin likely influenced by renal impairment. Inflammatory markers (CRP and fibrinogen) were elevated at admission and decreased by day 21, reflecting partial resolution with therapy. A notable aspect was the eosinopenia observed at admission in all patients, which resolved during treatment. This finding has been observed nonspecifically in severe bacterial infections and has even been proposed as a surrogate marker for sepsis [43,44].

Historically, hvKp strains have been described as broadly susceptible to antimicrobial agents, distinct from the MDR cKp strains often found in hospital settings. Our findings largely reflect this historical paradigm. All tested isolates demonstrated susceptibility patterns consistent with wild-type K. pneumoniae, with no acquired resistance to carbapenems, aminoglycosides, or fluoroquinolones detected in the majority of cases. The identification of a single MDR isolate (Case 4) exhibiting an ESBL-like phenotype is noteworthy. Although the isolate remained susceptible to carbapenems, and the specific resistance mechanism could not be confirmed due to a lack of molecular or phenotypic verification, this finding highlights a potential convergence of hypervirulence and antimicrobial resistance. While this finding does not indicate established dissemination, it should be regarded as a finding that warrants continued microbiological and genomic surveillance. The whole-genome sequencing data reveal five hvKp isolates (Cases 1, 9, 12, 14, and 15) as ST23-K1 with a high virulence potential (Kleborate score 5) and a consistent virulence gene repertoire (*rmp*A/A2, *mag*A, *peg-344*, and *iuc/iut/iro*). These determinants are likely carried by large IncFIB/IncHI1B plasmids.

**Figure 5:**
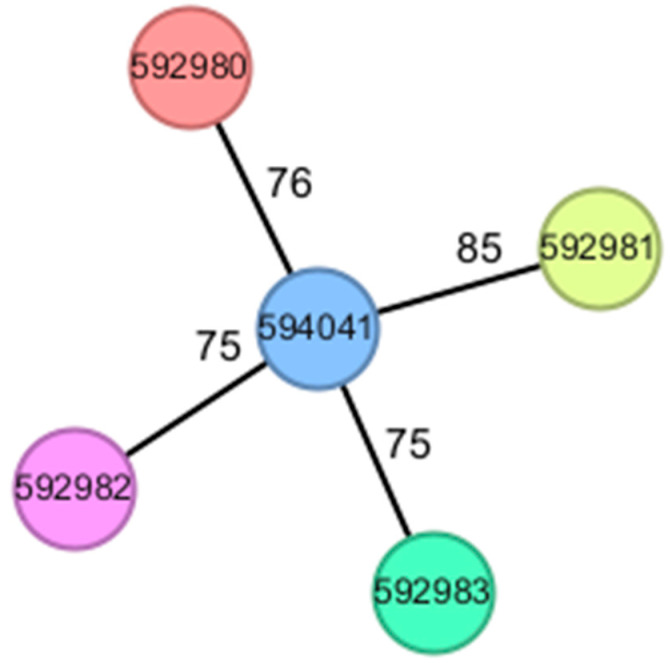
Minimum spanning tree based on cgMLST analysis (2358 loci) of the five ST23-K1 hypervirulent *Klebsiella pneumoniae* isolates. Each node represents a single isolate, colored according to complex type (CT). The numbers on the branches indicate allelic differences. The observed genetic distances support genetic heterogeneity among isolates, consistent with sporadic community acquisition rather than clonal transmission.

An important finding from the cgMLST analysis is the genetic heterogeneity among the sequenced isolates. Despite all belonging to the ST23 lineage, the isolates were assigned to distinct Cluster Types (CTs) and displayed significant distance on the minimum spanning tree (Figure 5). This diversity indicates that the cases in this cohort likely represent sporadic, community-acquired infections rather than a single clonal outbreak or nosocomial transmission chain. This suggests that highly virulent ST23-K1 strains are circulating in the community reservoir, independently infecting susceptible individuals rather than spreading from patient to patient within the healthcare facility.

### Limitations and Future Directions

This study is limited by the small sample size and the fact that WGS was only performed on a subset (*n* = 5) of the isolates. Consequently, we cannot definitively confirm the genetic basis of resistance in the non-sequenced MDR case (Case 4), although the phenotypic profile strongly suggests the presence of an ESBL gene (e.g., *bla*CTX-M). Future studies should aim to sequence all phenotypic variants to map the specific resistance mechanisms entering the hvKp population. Additionally, longitudinal surveillance is required to monitor for the potential acquisition of carbapenemase genes by these high-risk ST23 clones.

## 5. Conclusions

In this cohort of 15 patients with *Klebsiella pneumoniae* liver abscesses, type 2 diabetes was the predominant comorbidity. Despite severe complications such as endophthalmitis and septic emboli, the clinical outcomes were favorable in 14 cases following antibiotic therapy and drainage. Phenotypically, 93.3% of the isolates were wild-type susceptible, with only one ESBL-positive strain detected. Genomic analysis confirmed the presence of the hypervirulent ST23-K1 pandemic lineage carrying key virulence determinants (*magA, rmpA/A2, iuc,* and *peg-344*).

To our knowledge, these findings represent the first detailed molecular characterization of hypervirulent *K. pneumoniae* ST23-K1 in Romania and confirm the local circulation of high-risk clones with significant virulence potential. Although the present study did not directly assess transmission dynamics, admission screening strategies, or environmental contamination, the observed molecular profiles support the relevance of early molecular surveillance as part of broader infection prevention frameworks. Rigorous hygiene and environmental decontamination measures remain important considerations based on the existing evidence, in order to limit the dissemination of hypervirulent clones and reduce the risk of convergence with antimicrobial resistance.

We observed cases of liver abscesses caused by hypervirulent ST23-K1 *Klebsiella pneumoniae* in our setting. While the treatment response was generally good, severe disease and resistance were not absent. Awareness of these infections remains important in everyday clinical work.

## Figures and Tables

**Table 1:** The main characteristics of the 15 patients with liver abscesses caused by *Klebsiella pneumoniae*.

Case No.	Sex/Age	Main Comorbidities	Drainage Type	Complications	Duration of Antibiotic Therapy *	Outcome
1	M/74	CAD, BPH	Surgical (transferred to Thoracic Surgery)	Suspected PE	42 days	Favorable, size reduction
2	F/72	Type 2 DM, HTN, Parkinson’s disease	Percutaneous	Suspected septic pulmonary emboli	42 days	Favorable, size reduction
3	F/64	Type 2 DM, HTN, hTy	None	-	14 days	Favorable
4	F/74	Type 2 DM, renal lithiasis, DLP	None (transferred to Gastroenterology)	Sepsis	42 days	Favorable, size reduction
5	F/59	CAD, HTN, DLP, hTy	None (transferred to Cardiology)	Suspected PE	28 days	Favorable
6	M/69	HTN	None	Blepharoconjunctivitis	42 days	Favorable
7	M/74	HTN	None	-	35 days	Favorable
8	M/67	Type 2 DM, pancreatic neoplasm	None	-	28 days	Favorable
9	F/73	CAD, bronchiectasis, DLP	None	EE, septic shock	Death at 52 hours	Death
10	M/65	Type 2 DM, chronic ethanol use, DLP	Surgical	Associated SARS-CoV-2 infection	42 days	Favorable, size reduction
11	M/61	HTN	None	Hepatic vein thrombosis	28 days	Favorable, nearly normal imaging
12	M/64	Type 2 DM, ketoacidosis, DLP, hTy	None	EE	31 days	Favorable, size reduction
13	M/68	Type 2 DM, HTN	Percutaneous	Respiratory failure, anemia	21 days	Favorable, size reduction
14	M/44	Recurrent operated hernia	None	-	45 days	Favorable, size reduction
15	M/66	Type 2 DM, two prior episodes of liver abscesses	None	EE	45 days	Favorable, abscess remission

CAD = coronary artery disease, BPH = benign prostatic hyperplasia, hTy = hypothyroidism, PE = pulmonary embolism, DM = diabetes mellitus, HTN = arterial hypertension, DLP = dyslipidemia, EE = endogenous endophthalmitis, * = duration of antibiotic therapy during hospitalization. The cases for which the hvKp strains were sequenced are highlighted in gray.

**Table 2:** Main characteristics of hepatic imaging findings at admission.

Case No.	Hepatomegaly	Hepatic Steatosis Grade	Multiplicity	Maximum Dimensions (mm)	Localization (Segment)
1	Yes	II	Multiple	56 × 60	V-VI
2	No	II–III	Multiple	37 × 55	LSL
3	Yes	I	Single	73 × 64	VI
4	Yes	I	Multiple	57 × 60	VI-VII
5	Yes	I	Multiple	57 × 52	RHL
6	No	II	Multiple	30 × 29	BHL
7	Yes	Not specified	Single	36 × 21	VI
8	Yes	II	Multiple	57 × 52	VI-VII-VIII
9	Yes	II	Multiple	29 × 30	VI-VII-VIII
10	No	I	Multiple	140 × 100	V-VI-VII-VIII
11	Yes	I–II	Multiple	22 × 25	III- V-VII
12	Yes	I–II	Single	13 × 21	V
13	No	II–III	Single	77 × 54	VI
14	Not specified	Not specified	Multiple	47 × 46	II-VIII
15	Yes	I–II	Multiple	22 × 20	VI-VIII

LHL = left hepatic lobe, RHL = right hepatic lobe, BHL = both hepatic lobes; I–VIII represents the anatomical liver segment; sizes indicate the largest lesion documented by imaging. The cases for which the hvKp strains were sequenced are highlighted in gray.

**Table 3:** Main laboratory parameters at admission vs. day 21 of treatment.

Parameter	Admission (N = 15)				Day 21 (N = 12)			
	Mean	SD	Median	IQR	Mean	SD	Median	IQR
Leukocytes	13,660	6261	12,500	10,400	7525	2146	7000	2225
Monocytes	1074	590	1110	850	717	204	700	275
Neutrophils	11,586	5582	9100	8100	4817	1893	4200	1200
Eosinophils	9	27	0	0	200	191	150	200
Lymphocytes	927.33	608	800	855	1667	535	1550	700
Platelets	171,400	103,340	129,000	92,500	314,833	134,731	319,500	135,750
ALT	122.68	114.92	101	75	27	19	33	22
AST	115.68	106.85	87.75	138	23	6	26	6
Total Bilirubin	1	0.73	0.83	0.54	0.62	0.44	0.63	0.72
Direct Bilirubin	1.13	1.64	0.39	0.85	0.32	0.21	0.33	0.27
Creatinine	1.68	1.12	1.31	0.44	0.92	0.27	0.98	0.35
Fibrinogen	810	219	850	162	490	208	451	164
CRP (N ≤ 0.3 mg/dL)	24.52	9.77	23.6	19.75	2.59	2.84	0.77	4.84

Hematological parameters are expressed in cells/microliter; serum transaminase levels are reported in IU/mL (with normal values < 50 IU/mL); bilirubin, creatinine, and fibrinogen concentrations are presented in mg/dL.

**Table 4:** Molecular profile of sequenced hvKp strains.

Isolate ID (Sample Code)	592980	592981	592982	592983	594041
Capsular serotype	K1	K1	K1	K1	K1
MLST	ST23	ST23	ST23	ST23	ST23
cgMLST	CT17708	CT17709	CT3155	CT17710	CT17711
Main virulence genes	*magA, rmpA, rmpA2, rmpC, rmpD, iucABCD, iutA, iroBCDN, peg-344*	*magA, rmpA, rmpA2, rmpC, rmpD, iucABCD, iutA, iroBCDN, peg-344*	*magA, rmpA2, rmpC, rmpD, iucABCD, iutA, iroBCDN, peg-344*	*magA, rmpA, rmpA2, rmpC, rmpD, iucABCD, iutA, iroBCDN, peg-344*	*magA, rmpA2, rmpC, rmpD, iucABCD, iutA, iroBCDN, peg-344*
Kleborate virulence score	5	5	5	5	5
Resistance markers	*bla*SHV-1	*bla*SHV-1	*bla*SHV-1	*bla*SHV-1	*bla*SHV-1
Identified plasmids	IncFIB, IncH1B	IncFIB, IncH1B	IncFIB, IncH1B	IncFIB, IncH1B	IncFIB, IncH1B

## Data Availability

The corresponding author can provide the data used in this study upon reasonable request.

## Abbreviations

The following abbreviations are used in this manuscript:

ALT	alanine aminotransferase
AST	aspartate aminotransferase
BHL	both hepatic lobes
BMI	body mass index
BPH	benign prostatic hyperplasia
CAD	coronary artery disease
CAMHB	cation-adjusted Mueller–Hinton broth
cgMLST	core-genome multilocus sequence typing
cKp	classic *K. Pneumoniae*
CR-hvKp	carbapenem-resistant hypervirulent *Klebsiella pneumoniae*
CRP	C-reactive protein
CT	complex type
CT-exam	computer tomography examination
DLP	dyslipidemia
DM	diabetes mellitus
EE	endogenous endophthalmitis
EEA	European Economic Area
ENA	European Nucleotide Archive
ESBL	Extended-Spectrum Beta-Lactamase
EU	European Union
EUCAST	European Committee on Antimicrobial Susceptibility Testing
HTN	arterial hypertension
hvKp	hypervirulent *Klebsiella pneumoniae*
IgG	immunoglobulin G
IgM	immunoglobulin M
IQR	interquartile range
KLAs	*Klebsiella pneumoniae* liver abscesses
Kp	*Klebsiella pneumoniae*
KPC	*Klebsiella pneumoniae* carbapenemase
LHL	left hepatic lobe
MALDI-TOF MS	matrix-assisted laser desorption/ionization–time-of-flight mass spectrometry
MDR	multidrug-resistant
MIC	minimum inhibitory concentration
MLST	multilocus sequence typing
MST	minimum spanning tree
NDM	New Delhi metallo-*β*-lactamase
NIRDMI	National Institute of Research & Development for Microbiology & Immunology “Cantacuzino”
OXA-48	oxacillinase-48
PE	pulmonary embolism
RHL	right hepatic lobe
SD	standard deviation
ST	sequence type
T2DM	type 2 diabetes mellitus
VBH	“Dr. Victor Babes” Clinical Hospital of Infectious and Tropical Diseases
VFDB	Virulence Factor Database
VIM	Verona integron-encoded metallo-beta-lactamase
WGS	whole-genome sequencing
